# The influence and countermeasure of obesity in laparoscopic colorectal resection

**DOI:** 10.1002/ags3.12455

**Published:** 2021-05-13

**Authors:** Hideya Kashihara, Mitsuo Shimada, Kozo Yoshikawa, Jun Higashijima, Takuya Tokunaga, Masaaki Nishi, Chie Takasu, Toshiaki Yoshimoto

**Affiliations:** ^1^ Department of Surgery Tokushima University Tokushima Japan

**Keywords:** colorectal cancer, laparoscopic colorectal resection, obesity, preoperative weight loss program

## Abstract

**Background:**

The aim of this study was to investigate the influence of obesity and the usefulness of a preoperative weight loss program (PWLP) for obese patients undergoing laparoscopic colorectal resection (LCR).

**Methods:**

Study 1: 392 patients who underwent LCR for colorectal cancer were divided into two groups: those with a body mass index (BMI) ≥25 kg/m^2^ (n = 113) and those with a BMI <25 kg/m^2^ (n = 279). The influence of BMI on LCR was investigated. Study 2: Patients with a BMI ≥28 kg/m^2^ who were scheduled to undergo LCR (n = 7, mean body weight 87.0 kg, mean BMI 33.9 kg/m^2^) undertook a PWLP including caloric restriction and exercise for 29.6 (15–70) days. The effects of this program were evaluated.

**Results:**

Study 1: The BMI ≥25 kg/m^2^ group had a prolongation of operation time and hospital stay than the BMI <25 kg/m^2^ group. Study 2: The patients achieved a mean weight loss of 6.9% (−6.0 kg). The mean visceral fat area was significantly decreased by 18.0%, whereas the skeletal muscle mass was unaffected. The PWLP group had a significantly lower prevalence of postoperative complications compared with the BMI ≥25 kg/m^2^ group.

**Conclusion:**

Obesity affected the surgical outcomes in LCR. A PWLP may be useful for obese patients undergoing LCR.

## Abstract 2

This article indicated that preoperative weight loss program for obese patients undergoing laparoscopic colorectal resection was important. Our program is so useful for obese patients with colorectal cancer.

## 1 INTRODUCTION

The prevalence of obesity is currently very high in Western countries, and has also increased in Asian countries, including Japan.^1^ In laparoscopic surgery, obesity may reduce technical feasibility, prolong operative time, and increase operative blood loss. Therefore, obesity is a major risk factor for complications in laparoscopic surgery.^2^, ^3^, ^4^

Some authors have reported that laparoscopic colectomy can be safely performed in overweight and obese patients.^5^ However, others have reported that obese patients have a greater conversion rate to laparotomy, greater anastomotic leakage rate, and greater rate of complications compared with nonobese patients.^2^, ^6^ Furthermore, studies have reported that laparoscopic surgery for colorectal cancer is technically more difficult in obese patients than in nonobese patients.^2^, ^7^, ^8^

Previous studies have reported that obesity is a risk factor for complications after rectal surgery. Heus et al^9^ evaluated the influence of visceral obesity and muscle mass on postoperative complications in rectal surgery, and found that visceral obesity is correlated with a worse outcome after surgery for rectal cancer than body mass index (BMI), subcutaneous fat, and skeletal muscle area. Yamamoto et al^10^ reported that an increased BMI might be a potential risk factor for anastomotic leakage after laparoscopic surgery for rectal cancer using a stapling technique. Anastomotic leakage is reportedly associated with poor oncologic outcomes, especially regarding disease‐free survival.^11^

Therefore, obesity is an important risk factor of the severe complications and poor oncological outcomes after laparoscopic colorectal surgery. Thus, there is a need for preoperative intervention for obese patients with colorectal cancer.

Regarding preoperative weight loss before cancer surgery, Inoue et al^12^ reported that the preoperative 20‐day very low‐calorie diet weight loss program showed weight loss, reduction of visceral fat mass, and severe postoperative morbidity before laparoscopic gastrectomy for gastric cancer. In bariatric surgery, preoperative weight loss reduced the risk of postoperative complications and contributed to postoperative weight loss ^13^. However, there were only a few reports about preoperative weight loss in colorectal surgery. The effect of preoperative weight loss remains unclear, especially in cancer surgery.

To minimize these issues in obese patients undergoing laparoscopic colorectal resection (LCR), a preoperative weight loss program (PWLP) was started in our department. The aim of the present study was to investigate the influence of obesity on complications in LCR, and to investigate the usefulness of a PWLP for obese patients undergoing LCR.

## 2 METHODS

### 2.1 Study 1: The influence of BMI in laparoscopic anterior resection

From January 2007 to December 2015, 630 patients (open; n = 176, laparoscopy; n = 454) were referred to Tokushima University Hospital for the treatment of colorectal cancer. At first, 176 laparotomy cases were excluded. In 454 laparoscopy cases, the patients who underwent laparoscopic abdominoperineal resection (n = 38), Hartmann's operation (n = 4), total colectomy (n = 1), or temporary diverting stoma (n = 19) were excluded in this series. In total, 392 patients who underwent LCR with D2 or D3 lymph node dissection for colorectal cancer were enrolled in this study. All cancers were staged based on the Japanese Classification of Colorectal, Appendiceal, and Anal Carcinoma.^14^

The patients were divided into two groups: those with a BMI of ≥25 kg/m^2^ (BMI ≥25 kg/m^2^ group; n = 113) and those with a BMI of <25 kg/m^2^ (BMI < 25 kg/m^2^ group; n = 279). The patient background data are shown in Table 1.

**TABLE 1:** Patients' characteristics in BMI ≥25 and <25 groups in laparoscopic colorectal resection

Factors	BMI ≥ 25 (n = 113)	BMI < 25 (n = 279)	*P* value
Age (y.o.)	67.4 (37–85)	69.4 (34–92)	.10
Gender: Male/female	70/43	170/109	.85
BMI (kg/m^2^)	26.6 (25.0–41.8)	21.6 (13.1–24.9)	<.01
Tumor location (C/A/T/D/S/R)	17/5/10/7/30/44	50/20/12/14/81/102	.44
Operation type (IC/Partial/RHC/LHC/S/HAR/LAR)	17/17/5/0/30/12/32	50/27/16/3/81/19/83	.21
fStage: 0/I/II/III/IV/Others	5/34/32/32/6/4	11/83/73/74/22/16	.88

Note

The Mann‐Whitney *U*‐test was used for the statistical analysis of continuous variables, while the chi‐square test was used for categorical variables. *P* < .05 was considered to indicate a significant difference. Continuous variables are expressed as the mean ± standard deviation.

Abbreviation: BMI, body mass index.

### 2.2 Study 2: Evaluation of a PWLP for laparoscopic colorectal resection

**FIGURE 1:**
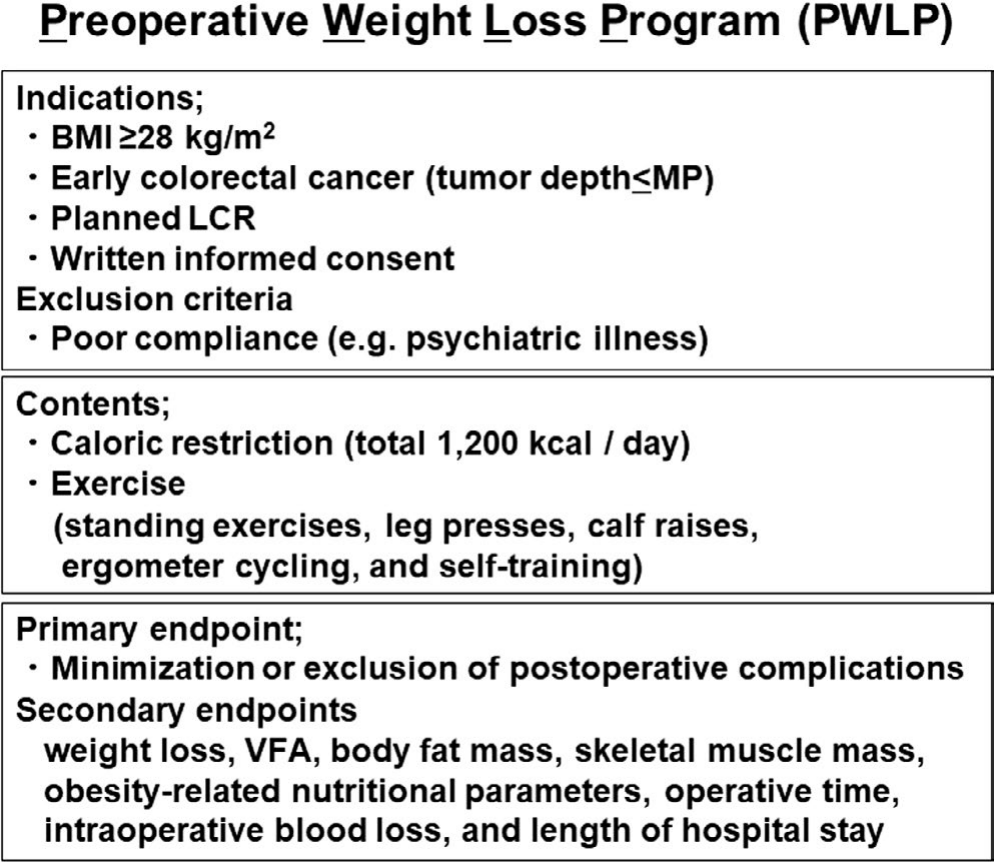
Preoperative weight loss program (PWLP)

To improve the operative outcomes in obese patients, a PWLP was started in the Department of Surgery, Tokushima University, with the approval of a suitably constituted Ethics Committee of Tokushima University Hospital (Figure 1).

The indications for a PWLP at Tokushima University Hospital were a BMI ≥28 kg/m^2^, early colorectal cancer (tumor depth ≤muscularis propria), and a planned LCR. Written informed consent was provided by each patient before surgery. The exclusion criteria were conditions associated with poor compliance (e.g., psychiatric illness). There were no dropped out patients in this study.

The PWLP comprised caloric restriction (total 1200 kcal per day) and exercise (matched age and activities of daily living) for 29.6 (15–70) days. The exercise in the PWLP comprised standing exercises, leg presses, calf raises, ergometer cycling, and self‐training in the Department of Rehabilitation.

The primary endpoint was the minimization or exclusion of postoperative complications. The secondary endpoints were: weight loss, visceral fat area (VFA), body fat mass, skeletal muscle mass, obesity‐related nutritional parameters, operative time, intraoperative blood loss, and length of hospital stay. The data regarding VFA, body fat mass, and skeletal muscle mass were obtained by the InBody 770 (Cerritos, CA).

Table 2 shows the characteristics of the patients in the PWLP. The PWLP participants were patients with a BMI of ≥28 kg/m^2^ who were scheduled to undergo LCR (n = 7, mean body weight 87.0 kg, mean BMI 33.9 kg/m^2^). These PWLP participants were enrolled from 2016 to 2019. To confirm the tumor progression in PWLP, we checked tumor marker, computed tomography, and colonoscopy in pre‐ and post‐PWLP. There was no tumor progression in all patients who underwent PWLP.

**TABLE 2:** Patient characteristics in the PWLP group

Factors	n = 7
Age (y.o.)	65.7 (53–80)
Gender: Male/female	3/4
BMI (kg/m^2^)	33.9 (30.7–43.7)
Body weight (kg)	87.0 (73.9–109)
Comorbidity (+/−)	4/3
Duration of PWLP (days)	29.6 (15–70)
Tumor location (A/T/D/S/R)	1/1/1/3/1
Operation type (IC/partial resection/HAR)	1/4/2
fStage (I/II)	6/1

Note

Continuous variables are expressed as the mean ± standard deviation.

Abbreviation: PWLP, preoperative weight loss program.

General clinical and clinicopathological data from each eligible patient were retrieved from the medical reports. All data were reviewed retrospectively, and the Clavien‐Dindo classification was used to evaluate the short‐term complication rate during hospitalization.

### 2.3 Statistical analysis

The unpaired/paired Student's *t*‐test or the Mann‐Whitney *U*‐test was used for the statistical analysis of the continuous variables, while the chi‐square test was used for the categorical variables. In all three statistical tests, *P* < .05 was considered to represent a significant difference. The values for each of the continuous variables are expressed as the mean ± standard deviation.

To test the independence of risk factor for postoperative complication and anastomotic leakage, all factors in univariate analyses were included in a final model of logistic regression. All statistical analyses were performed using JMP software (SAS Institute, Cary, NC).

## 3 RESULTS

### 3.1 Study 1: The influence of BMI in laparoscopic anterior resection

Table 3 shows the operation times and intraoperative blood loss in the BMI ≥25 kg/m^2^ and BMI <25 kg/m^2^ groups. The operation time of the BMI ≥25 kg/m^2^ group was significantly longer than that of the BMI <25 kg/m^2^ group. Postoperative complications of Clavien‐Dindo grade II and anastomotic leakage did not significantly differ between the two groups. The duration of postoperative hospitalization of the BMI ≥25 kg/m^2^ group was significantly longer than that of the BMI <25 kg/m^2^ group.

**TABLE 3:** Operative outcomes in BMI ≧25 and <25 groups in laparoscopic colorectal resection

Factors	BMI ≥ 25 (n = 113)	BMI < 25 (n = 279)	*P* value
Operation time (min)	231.0 (102–510)	207 (50–551)	<.05
Blood loss (mL)	20.0 (0–650)	20 (0–2260)	.84
Postoperative complications (CD ≥ grade 2)	14.2%	12.9%	.74
	Ileus (n = 4, 3.5%)	Ileus (n = 8, 2.9%) Ascites (n = 4, 1.4%)	.26
Anastomotic leakage	8.0%	5.0%	.26
Hospital stay (days)	23.6 (6–382)	17.6 (6–85)	<.05

Note

The Mann‐Whitney *U*‐test was used for the statistical analysis of continuous variables. *P* < .05 was considered to indicate a significant difference. Continuous variables are expressed as the mean ± standard deviation.

Abbreviation: BMI, body mass index.

Table 4 shows the comparison of with and without postoperative complication and anastomotic leakage in laparoscopic colorectal resection. In the complication group, there was a significantly higher prevalence of rectum, operation time ≥300 minutes, and blood loss ≥100 mL. In the anastomotic leakage group, there was a significantly higher prevalence of rectum and operation time ≥300 minutes were independent risk factors of anastomotic leakage. Regarding postoperative complication, blood loss ≥100 mL was an independent risk factor in multivariate analysis. Furthermore, rectum and operation time 300 ≥minutes were independent risk factors of anastomotic leakage (Table 5).

**TABLE 4:** Comparison of with and without postoperative complication and anastomotic leakage in laparoscopic colorectal resection

Factors	Complications (n = 52)	No complications (n = 340)	*P* value	Anastomotic leakage (n = 23)	No anastomotic leakage (n = 369)	*P* value
Age (y.o.): <70/70≤	27/25	183/157	.80	16/7	194/175	.11
Gender: Male/female	37/15	203/137	.11	18/5	222/147	.08
BMI (kg/m^2^): <25/25≤	36/16	243/97	.74	14/9	265/104	.26
Tumor location: Colon/rectum	24/28	222/118	<.01	6/17	240/129	<.01
Operation time (min): <300/300≤	38/14	301/39	<.01	15/8	323/46	<.01
Blood loss (mL): <100/100≤	39/13	303/37	<.01	21/2	321/48	.59

Note

The chi‐square test was used for categorical variables. *P* < .05 was considered to indicate a significant difference.

Abbreviation: BMI, body mass index.

**TABLE 5:** The independent risk factor of postoperative complications and anastomotic leakage

Factors	Postoperative complications		Anastomotic leakage	
	Hazard ratio	*P* value	Hazard ratio	*P* value
Age (y.o.): 70≤	0.41	.69	−1.29	.20
Gender: Male	1.17	.24	1.17	.24
BMI (kg/m^2^): 25≤	0.46	.65	0.94	.35
Tumor location: Rectum	1.63	.11	2.32	<.05
Operation time (min): 300≤	1.70	.09	2.27	<.05
Blood loss (mL): 100≤	2.28	<.05	−0.99	.32

Note

To test the independence of risk factor for postoperative complication and anastomotic leakage; all factors in univariate analyses were included in a final model of logistic regression.

Abbreviation: BMI, body mass index.

### 3.2 Study 2: Evaluation of a PWLP for laparoscopic colorectal resection

3.2

The patients achieved a mean weight loss of 6.9% (−6.0 kg) after the PWLP. Compared with the pre‐PWLP values, the mean post‐PWLP VFA and body fat mass were significantly decreased (mean VFA decrease 18.0%; mean body fat mass decrease 9.3%), whereas the skeletal muscle mass was unchanged.

Regarding the obesity‐related parameters, the mean total cholesterol was significantly decreased after the PWLP. However, the mean pre‐ and post‐PWLP albumin values did not significantly differ (Table 6).

**TABLE 6:** Obesity‐related factors in pre‐ and post‐PWLP

Factors	Pre‐PWLP	Post‐PWLP	*P* value
Body weight (kg)	87.0 (73.9–109)	81.0 (69.8–96.0)	<.05
BMI (kg/m^2^)	33.9 (30.7–43.7)	31.6 (28.9–38.0)	<.05
Visceral fat area (m^2^)	169.0 (114.5–217.0)	138.6 (91.5–212.0)	<.05
Body fat mass	40.7% (30.0–54.1)	36.9% (26.2–47.8)	<.05
Skeletal muscle	31.8% (21.9–48.7)	31.1 (21.8–44.6)	.26
AST (IU/L)	27.1 (14–45)	23.7 (13–39)	.53
ALT (IU/L)	24.9 (8–39)	18.4 (9–26)	.21
Total cholesterol (mg/dL)	214.9 (189–258)	167.8 (116–188)	<.05
Albumin (g/dL)	4.1 (3.6–4.4)	4.0 (3.6–4.3)	.39

Note

The Mann‐Whitney *U*‐test was used for the statistical analysis of continuous variables. *P* < .05 was considered to indicate a significant difference.

Abbreviation: PWLP, preoperative weight loss program.

Table 7 shows the comparison of operative outcomes between the BMI ≥25 and PWLP group. In the BMI ≥25 group of Table 7, the cases of fStageIII, IV (n = 38) were excluded to match the tumor progression. The PWLP group showed a lower prevalence of postoperative complications (including anastomotic leakage) compared with the BMI ≥25 kg/m^2^ group. There was no significant difference in operation time and postoperative hospital stay between the two groups (Table 7).

**TABLE 7:** Comparison of operative outcomes between the BMI >25 and PWLP group

Factors	BMI >25^a^ (n = 75)	PWLP (n = 7)	*P* value
Operation time (min)	231.5 (102–510)	237.9 (159–305)	.92
Postoperative complication (CD > grade 2)	16.0%	0%	<.05
Anastomotic leakage	8.0%	0%	<.05
Hospital stay (days)	14.0 (6–382)	15.0 (9–28)	.56

Note

The Mann‐Whitney *U*‐test was used for the statistical analysis of continuous variables. *P* < .05 was considered to indicate a significant difference.

Abbreviations: BMI, body mass index; PWLP, preoperative weight loss program.

^a^ Excluded the cases of fStage III, IV (n = 38).

## 4 DISCUSSION

The present study was designed to investigate the influence of obesity on LCR for colorectal cancer, and to evaluate the usefulness of a PWLP for obese patients undergoing LCR for colorectal cancer. The BMI ≥25 kg/m^2^ group had a significantly longer operation time and postoperative hospital stay than the BMI <25 kg/m^2^ group. The PWLP group achieved significant decreases in body weight and VFA. Furthermore, the PWLP group had a lower prevalence of postoperative complications compared with the BMI ≥25 kg/m^2^ group.

For patients undergoing laparoscopic sleeve gastrectomy, a preoperative diet comprising immune‐enhancing nutrition formulas reportedly achieves greater preoperative weight loss, lower postoperative pain, and lower levels of C‐reactive protein and liver enzymes than high‐protein formulas or a regular diet with similar caloric intakes.^15^ However, one of the important problems with weight loss is the incidence of sarcopenia. A decrease in skeletal muscle, referred to as sarcopenia, is reportedly correlated with morbidity and mortality in patients undergoing digestive surgery. Thus, unintentional weight loss can be used to predict mortality and morbidity rates in colorectal surgery. In particular, preoperative weight loss is significantly associated with cardiopulmonary complications.^16^ Kaido et al^17^ reported that sarcopenia is closely related with postoperative mortality in patients undergoing living donor liver transplantation and that perioperative nutritional intervention is important for the survival of patients with sarcopenia. Sarcopenic obesity is defined using the criteria for sarcopenia and obesity by body fat mass (≥25% body fat for males, ≥35% body fat for females). In patients undergoing surgery for hepatocellular carcinoma, preoperative sarcopenic obesity is an independent risk factor for patient survival and postoperative recurrence.^18^

In preoperative weight loss, the prevention of sarcopenia is very important in decreasing perioperative complications. In the present study, the PWLP resulted in the maintenance of several nutritional parameters and did not cause a decrease in skeletal muscle mass. Yamamoto et al^19^ reported that preoperative exercise and a nutritional support program for elderly sarcopenic patients with gastric cancer increased handgrip strength, gait speed, and skeletal muscle mass index. The intervention of nutrition and exercise was essential for the maintenance of skeletal muscle.

Patients with both visceral obesity and sarcopenia were reported to have a higher complication rate after colorectal cancer surgery.^20^ In this report, age ≥65 years, visceral obesity, and sarcopenia were independent risk factors for total complications. Obesity with reduced muscle mass was related to higher 30‐day morbidity and mortality rates.^21^ In addition, postoperative complication, especially anastomotic leakage, was reported to be associated with poor oncologic outcomes.^11^ Sarcopenia and fatty infiltration of the muscle (myosteatosis) were independent predictors of worse survival in Stage I to III colorectal cancer. And their joint effect was highly reduced oncologic survival.^22^ Therefore, obesity and sarcopenia are associated with difficulty of surgical procedure, postoperative complication, and poor postoperative oncological outcomes. Thus, the preoperative intervention for obesity and sarcopenia is very important for improving short‐ and long‐term surgical outcomes.

The progression of colorectal cancer during the PWLP may be a major problem. However, Brenkman et al^23^ reported that a longer waiting time (even >8 weeks) before gastrectomy surgery for gastric cancer is not associated with a worse overall survival rate. In addition, Fujiya et al^24^ also reported that a prolonged wait time (6 mo) for surgery was not related with survival of Stage I gastric cancer. Therefore, the duration of the PWLP may be reasonable for the purpose of decreasing postoperative complications.

The limitations of this study were that it was a retrospective study, single institution, and a small number of patients. In addition, the comparison between the PWLP group and the no‐PWLP group in the same background was not done.

In conclusion, obesity affected the operative outcomes in LCR. A PWLP may be useful for obese patients undergoing LCR, and a PWLP may be useful in minimizing complications for obese patients undergoing LCR.

## DISCLOSURE

Conflict of Interest: Hideya Kashihara and all other co‐authors declare no conflicts of interests regarding this article. The protocol for this research project has been approved by a suitably constituted Ethics Committee of the institution and it conforms to the provisions of the Declaration of Helsinki. Committee of Tokushima University Hospital, Approval No. R000030188. Informed consent was obtained from all subjects.
